# A United States HIV provider survey of antiretroviral therapy management in people living with HIV with co-occurring conditions

**DOI:** 10.1186/s12981-025-00724-w

**Published:** 2025-03-01

**Authors:** Sonya Krishnan, Marina B. Martinez Rivera, Christopher K. Lippincott, Maunank Shah

**Affiliations:** 1 Department of Medicine, Division of Infectious Diseases, Johns Hopkins University School of Medicine, Baltimore, MD USA; 2 1550 Orleans St, CRB Building, 1M-10, Baltimore, MD 21287 USA

**Keywords:** HIV, Non-infectious comorbidities, Management strategies, Antiretroviral therapy

## Abstract

**Introduction:**

Simplified HIV treatment guidelines favor integrase strand transfer inhibitors (INSTIs). However, non-infectious comorbidities and co-occurring conditions (i.e. pregnancy) often necessitate individualized antiretroviral therapy (ART) regimens. This study aimed to characterize United States HIV provider strategies for ART selection when faced with concomitant health conditions.

**Methods:**

A survey of US HIV providers was conducted using hypothetical patient cases. Standardized clinical case-vignettes were developed and providers were asked to select their preferred regimen. Eleven cases focused on cardiometabolic syndrome, renal dysfunction, weight gain, and pregnancy.

**Results:**

119 providers responded across all cases (with a median 57 responses [interquartile range 55.5–72] per case), and were primarily Infectious Diseases physicians in academic settings from across the continental United States. Bictegravir/tenofovir alafenamide/emtricitabine was most commonly prescribed for three case-scenarios of cardiometabolic disease (62.3%). Diverse regimens were recommended for a case involving weight gain, with 98.5% switching from dolutegravir plus tenofovir alafenamide/emtricitabine, most commonly to doravirine/tenofovir disoproxil fumarate/lamivudine. Dolutegravir-based regimens were selected in case-scenarios of pregnancy (77.3%), with some use of bictegravir/tenofovir alafenamide/emtricitabine (13.6%). For two case-scenarios renal disease with worsening creatinine clearance to < 30 mL/minute, many providers used lamivudine or emtricitabine in fixed-dose combination (43.3%).

**Conclusion:**

This study reveals varied ART approaches for people living with HIV and non-infectious conditions, often diverging from standard regimens. While guidelines provide a framework, providers adapt treatment based on patient needs. Further research is crucial to optimize ART management in these complex situations.

**Supplementary Information:**

The online version contains supplementary material available at 10.1186/s12981-025-00724-w.

## Background

Global guidelines for HIV management have continued to simplify, with integrase-strand transfer inhibitors (INSTIs) favored as anchor drugs in initiating antiretroviral therapy (ART) regimens [1–3]. In the United States (US), current guidelines by the International Antiviral Society-USA (IAS-USA) and the Department of Health and Human Services (DHHS) recommend initiating either dolutegravir with tenofovir alafenamide (TAF) and emtricitabine, bictegravir/TAF/emtricitabine, or in some circumstances dolutegravir/lamivudine for ART-naïve patients [2, 3]. While the widespread availability of effective ART has dramatically prolonged life expectancies for people living with HIV (PLHIV), the incidence of non-infectious conditions, such as cardiovascular disease, renal disease, and diabetes, remains high as the population ages [4, 5].

In PLHIV with pregnancy or certain comorbidities, such as obesity, renal dysfunction, cardiovascular disease, use of first line ART regimens requires careful consideration, as data has potentially implicated ART worsening the underlying comorbidity [6, 7]; or in the case of pregnancy ART has not been well-studied or is associated with fetal toxicity [8]. For these scenarios, US guidelines advocate for an individualized ART approach that accounts for multiple patient characteristics, but the guidelines can be complex and difficult to navigate. Furthermore, providers may not always implement updated guidelines or guidelines may lag behind provider practice.

We previously presented the results of 16 cases involving ART resistance and found general consensus in the approach to cases with single class resistance, but a wide variety of practice as drug-class resistance increased [9]. Clinical practice patterns among US HIV providers managing non-infectious comorbidities or co-occurring conditions have not been well characterized. We therefore used hypothetical case-scenarios involving PLHIV with comorbidities or pregnancy, to ascertain ART management strategies for cardiometabolic syndrome, renal dysfunction, weight gain, and pregnancy.

## Materials and methods

### Participant selection

Our study methodology has previously been detailed [9]. In brief, HIV providers throughout the continental US were identified using professional network societies and contacts at academic institutions. Providers were emailed a Qualtrics e-survey and responded from August 26, 2022 to December 11, 2022.

### Standardized case scenarios

**Fig. 1:**
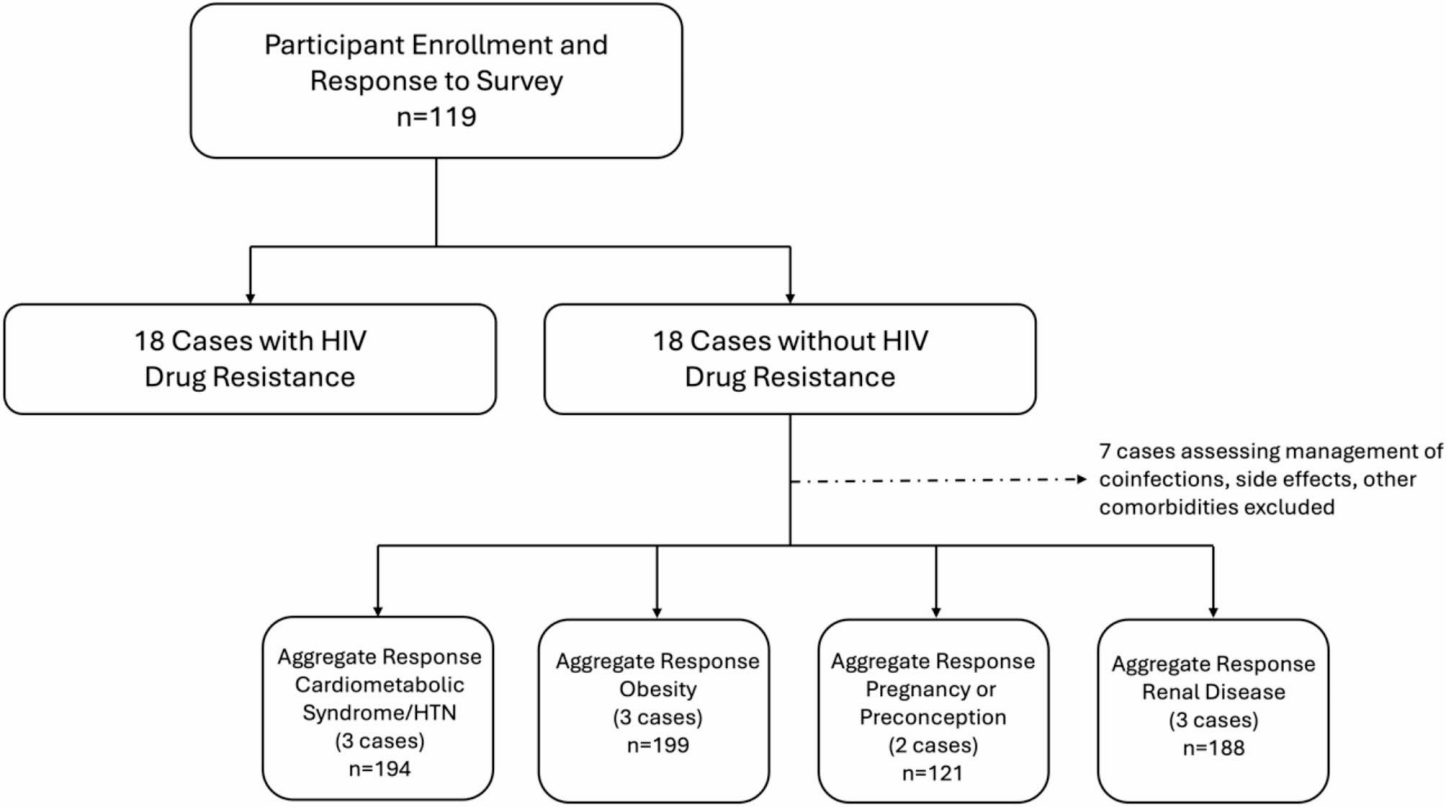
Study flowchart of responses of providers (MD/DO, advanced practice provider, pharmacists) with experience caring for people living with HIV. Participating individuals completed the survey of 2 blocks of 6 cases with and without HIV resistance. They were invited to complete additional cases

We developed 36 hypothetical, standardized case-vignettes – the scenarios focused either on an approach to HIV resistance or accompanying comorbidities or co-occurring conditions. Participants were asked to propose their single preferred ART regimen. Participants completed an initial survey of six randomly selected case-vignettes. They had the option to complete up to 36 total cases. We now present the results from 11 cases involving new or progressive conditions, grouped by cardiometabolic syndrome, renal disease, weight gain, and pregnancy (Fig. 1). Full case-vignettes details are available in Supplementary Table 2.

### Data collection and statistical analyses

Data was aggregated using Qualtrics and descriptive statistics were used to detail participant demographics and the frequency of selected regimens (StataCorp, College Station, TX, Version 17.0). Incomplete responses were excluded (i.e. blank, monotherapy).

## Results

### Participant characteristics

119 providers participated in the survey and were mostly physicians (*n* = 87; 73.1%) with 10 + years of clinical experience (*n* = 51; 42.9%), from Infectious Diseases specialty (*n* = 98; 82.4%), and practiced in an Academic/University setting (*n* = 108; 90.8%), who spent either < 25% (*n* = 41; 34.5%) or 25–50% (*n* = 37; 31.1) of their time caring for PLHIV. The practiced across all regions of the continental US (Top 3 Regions: Mid-Atlantic (*n* = 32; 26.9%), Midwest (*n* = 30; 25.5%), West (*n* = 19; 16.0%). Full participant characteristics were previously reported [9]. There was a median of 57 (interquartile range [IQR] 55.5–72) responses per case, across 11 cases involving comorbidities (Fig. 1).

### Cardiometabolic syndrome, hypertension, and hyperlipidemia cases

Across three case-scenarios (C1, C2, C3) of patients newly diagnosed with HIV with cardiometabolic syndrome, hypertension and/or hyperlipidemia, the two most prescribed regimens were bictegravir/TAF/emtricitabine (*n* = 119; 62.3%) and dolutegravir/lamivudine (*n* = 33; 17.3%) (Table 1). 9.4% (*n* = 18) selected a 3-drug regimen consisting of a non-nucleoside reverse transcriptase inhibitor (NNRTI)/tenofovir disoproxil fumarate (TDF)/lamivudine or emtricitabine, and 14.7% (*n* = 28) selected an INSTI-sparing regimen (Supplementary Table 3).

**Table 1:** Summary of responses by case scenario

Case	Case Summary	Comorbidities, Conditions & Comedications	*N*	Summary of Most Common Response^a, b^ Regimen *n* (%)				Current US Guideline Recommendation
C1	72 yo M, new HIV diagnosis CD4 450, HIV VL 55 K	*Comorbidities*: Coronary Artery Disease, Type II Diabetes (T2DM), Hyperlipidemia, Obesity (body mass index [BMI] 31.1 kg/m2) *Comedications*: Aspirin, Lisinopril, Metoprolol, Metformin, Rosuvastatin	80	BIC/TAF/FTC DTG/3TC DOR/TDF/3TC	50 13 7	(62.5%) (16.3%) (8.8%)		*DHHS*: ABC and PI associated with cardiovascular disease in some studies. TDF associated with less hyperlipidemia *IAS-USA*: Switch off of ABC-containing regimen. *HIVMA/IDSA*: Statin initiation, TDF associated with less hyperlipidemia All sections without specific ART recommendations besides ABC.
C2	58 yo M, new HIV diagnosis CD4 174, HIV VL 81 K	*Comorbidities*: Obesity (BMI 31 kg/m^2^), Non-alcoholic Steatohepatitis, Prediabetes, Hypertriglyceridemia *Comedications*: None	54	BIC/TAF/FTC DTG/3TC DOR/TDF/3TC DRV/c/TAF/FTC	22 12 10 4	(40.7%) (22.2%) (18.5%) (7.4%)		
C3	70 yo M, new HIV diagnosis CD4 189, HIV VL 79 K	*Comorbidities*: HTN, Hyperlipidemia *Comedications*: Losartan, Hydrochlorothiazide, Amlodipine, Simvastatin	57	BIC/TAF/FTC DTG/3TC	47 8	(82.5%) (24.0%)		
O1	39 yo F, new HIV diagnosis CD4 450, HIV VL 34 K Patient expresses concern for weight gain on antiretrovirals	*Comorbidities*: Obesity (BMI 29 kg/m^2^) *Comedications*: Oral Combined Hormonal Contraceptive	79	DOR/TDF/3TC RPV/TAF/FTC BIC/TAF/FTC DRV/c/TAF/FTC DTG/3TC DTG/ABC/3TC	14 13 11 9 9 6	(17.7%) (16.5%) (13.9%) (11.4%) (11.4%) (7.6%)		*DHHS*: Concern for weight gain is not a reason to avoid INSTI-based regimen for initial ART. Flexibility to assess risks and benefits of regimens when changing ART, especially in Black women *IAS-USA*: Changing regimens because of weight gain not recommended due to TDF toxicity and lack of benefit in switch from INSTI to PI *HIVMA/IDSA*: Current data do not support switching an ART regimen solely for weight gain
O2	58 yo F, 15-year history of HIV CD4 650, HIV VL < 20 On DRV/c/TAF/FTC 20-pound weight gain over last 2 years	*Comorbidities*: Type II diabetes, Hypertension (HTN), Hyperlipidemia, Obesity (BMI 30 kg/m^2^) *Comedications*: Metformin, Amlodipine, Losartan, Rosuvastatin	57	DRV/c/TAF/FTC DOR/TDF/3TC CAB/RPV DTG/3TC BIC/TAF/FTC	13 12 11 8 5	(22.8%) (21.1%) (19.3%) (14.0%) (8.8%)		
O3	52 yo M, well-controlled HIV CD4 > 200, HIV VL < 20 30-pound weight gain over last 3 years on DTG + TAF/FTC	*Comorbidities*: Obesity (BMI 31.6 kg/m^2^) *Comedications*: None	66	DOR/TDF/3TC DRV/c/TAF/FTC DTG/3TC RPV/TAF/FTC RPV/TDF/FTC	17 11 7 6 4	(25.8%) (16.7%) (10.6%) (9.1%) (6.1%)		
P1	31 yo F, newly diagnosed HIV CD4 195, HIV VL 250 K	*Co-occurring Condition*: Pregnant in 1st Trimester *Comedications*: None	66	DTG, TAF/FTC DTG/ABC/3TC DTG, TDF/FTC BIC/TAF/FTC	22 14 12 9	(33.3%) (21.2%) (18.2%) (13.6%)		DHHS: Shared decision-making emphasized. TDF, TAF, XTC, DTG preferred. BIC, DRV/r, oral RPV, EFV alternative *IAS-USA*: TAF/XTC + DTG preferred, TDF/XTC alternative. DRV/r, BIC/TAF/FTC alternative. RPV insufficient data. Switch off of long-acting injectable
P2	31 yo F, treatment naïve CD4 195, HIV VL 250 K	*Comorbidities*: Preconception, desires pregnancy *Comedications*: None	53	DTG, TAF/FTC DTG/ABC/3TC BIC/TAF/FTC DTG, TDF/FTC	15 12 9 6	(28.3%) (22.6%) (17.0%) (11.3%)		
R1	46 yo M, newly diagnosed HIV CD4 > 200, HIV VL 100 K	*Comorbidities*: End Stage Renal Disease on Hemodialysis, HTN *Comedications*: Erythropoetin, Vitamin D, Sevelamer, Iron, Atenolol, Amlodipine	54	BIC/TAF/FTC DTG/3TC DTG/ABC/3TC EVG/c/TAF/FTC	21 5 4 4	(38.9%) (9.3%) (7.4%) (7.4%)		*DHHS*: Insufficient evidence for or against full-dose 3TC or FTC with CrCl < 30 mL/min. TAF not recommended for CrCl < 15 mL/min. *IAS-USA*: Switches may be made, but no specific regimen or dosing recommendations
R2	58 yo M, longstanding HIV CD4 > 200, HIV VL < 20 On BIC/TAF/FTC Worsening renal function	*Comorbidities*: Chronic Kidney Disease (CrCl 30–59 mL/min) now with > 25% eGFR decline and 1 + proteinuria, HTN, T2DM, Depression *Comedications*: Sertraline, Lisinopril, Metformin	77	DTG/RPV CAB/RPV DTG/3TC DTG/ABC/3TC BIC/TAF/FTC	24 12 11 9 8	(31.2%) (15.6%) (14.3%) (11.7%) (10.4%)		
R3	54 yo F, longstanding HIV CD4 > 200, HIV VL < 20 On BIC/TAF/FTC Worsening renal function	*Comorbidities*: Chronic Kidney Disease Stage 4 with decline in CrCl from 45 mL/min to < 30 mL/min *Comedications*: None	57	DTG/3TC CAB/RPV DTG/RPV BIC/TAF/FTC	18 15 12 10		(31.6%) (26.3%) (21.1%) (17.5%)	

Abbreviations: M, Male; HIV, Human Immunodeficiency Virus; VL, viral load; K, Thousand; F, Female; CrCl, creatinine clearance; eGFR, estimated Glomerular Filtration Rate, DHHS, Department of Health and Human Services [2]; IAS-USA, International Antiviral Society–USA [3]; HIVMA/IDSA, HIV Medicine Association of the Infectious Diseases Society of America [10]

^a^For a list of antiretroviral abbreviations, full clinical vignette details, as well as full antiretroviral regimen responses, see Supplementary Tables 1–3

^b^Regimens with less than *n* = 4 responses not displayed

### Documented weight gain or concern for weight gain

In case O1 of a female patient newly diagnosed with HIV who expressed concern for weight gain and was overweight (body mass index 29 kg/m^2^), the most prescribed regimens were doravirine/TDF/lamivudine (*n* = 14, 17.7%), rilpivirine/TAF/emtricitabine (*n* = 13, 16.5%), and bictegravir/TAF/emtricitabine (*n* = 11, 13.9%). In case O2 of a patient with a 20-pound weight gain over the last two years on darunavir/cobicistat/TAF/emtricitabine, the most commonly selected regimens were continuation of darunavir/cobicistat/TAF/emtricitabine (*n* = 13, 22.8%), or switches to doravirine/TDF/lamivudine (*n* = 12, 21.1%) and cabotegravir/rilpivirine (*n* = 11, 19.1%) (Table 1). Overall, 24.6% (*n* = 14) switched to a regimen containing TDF and 38.6% (*n* = 22) switched to a tenofovir-sparing regimen. In case O3 of a patient with a 30-pound weight gain on an INSTI and TAF (dolutegravir + TAF/emtricitabine), there were 18 different regimens recommended (Supplementary Table 3). The most common regimens were doravirine/TDF/lamivudine (*n* = 17, 25.8%) and darunavir/cobicistat/TAF/emtricitabine (*n* = 11, 16.7%). Only 1.5% (*n* = 1) maintained the regimen, while 37.9% (*n* = 25) selected an INSTI/TAF-sparing regimen; 24.2% (*n* = 16) selected a tenofovir-sparing regimen (Supplementary Table 3).

### Pregnancy and preconception

Case P1 featured a 1st trimester pregnant ART-naïve woman newly diagnosed with HIV. The most common regimens initiated contained dolutegravir (*n* = 51, 77.3%), however, a subset of providers started bictegravir/TAF/emtricitabine (*n* = 9, 13.6%) (Table 1). 53.0% (*n* = 35) of regimens contained TAF and 25.8% (*n* = 17) contained TDF. 4.5% (*n* = 3) initiated the patient on a protease inhibitor (PI). Similarly, in a case P2 of a treatment naïve woman living with HIV expressing desire for pregnancy, 73.6% (*n* = 39) began a dolutegravir-containing regimen and 17.0% (*n* = 9) selected bictegravir/TAF/emtricitabine (Table 1). 56.6% (*n* = 30) of regimens contained TAF where as 17.0% (*n* = 9) contained TDF; 3.8% (*n* = 2) initiated a PI (Supplementary Table 3).

### Renal disease

In case R1 of an individual with end stage renal disease (ESRD) on hemodialysis and newly diagnosed HIV, the most frequently prescribed regimen was bictegravir/TAF/emtricitabine (*n* = 21, 38.9%) (Table 1). 38.9% (*n* = 21) selected a tenofovir-sparing regimen and 20.4% (*n* = 11) selected a regimen that contained lamivudine in FDC (Supplementary Table 3). In case R2 of a patient with chronic kidney disease (CKD; creatinine clearance [CrCl] 30–59 mL/minute) on bictegravir/TAF/emtricitabine who developed > 25% estimated glomerular filtration rate (eGFR) decline and 1 + proteinuria, providers most often switched to tenofovir-sparing regimens such as dolutegravir/lamivudine (*n* = 18, 31.6%), cabotegravir/rilpivirine (*n* = 15, 26.3%) or dolutegravir/rilpivirine (*n* = 12, 21.1%); alternatively, 17.5%( *n* = 10 ) maintained the patient on bictegravir/TAF/emtricitabine (Table 1). 82.5% (*n* = 47) selected a tenofovir-sparing regimen and 50.9% (*n* = 29) selected a FDC that contained lamivudine or emtricitabine (Supplementary Table 3). In case R3 of a patient with longstanding HIV and a history of CKD on bictegravir/TAF/emtricitabine who developed worsening renal function and a CrCl of < 30 mL/minute, providers most often switched to dolutegravir/rilpivirine (*n* = 24, 31.2%), cabotegravir/rilpivirine (*n* = 12, 15.6%), or dolutegravir/lamivudine (*n* = 11, 14.3%) (Table 1). 88.3% (*n* = 68) selected a tenofovir-sparing regimen and 37.7% (*n* = 29) selected a regimen that contained lamivudine or emtricitabine in FDC (Supplementary Table 3).

## Discussion

Our study of HIV providers throughout the US highlights offers insight to the approach to ART management and comorbidities or co-occuring conditions, where providers often make decisions based on individual patient needs and preferences. In particular, in case O3 of weight gain on dolutegravir plus TAF/emtricitabine, 98.5% (*n* = 65) of providers switched regimens despite limited current evidence that a switch would curb or reverse weight gain; new HIV Medical Association/Infectious Diseases Society of America (HIVMA/IDSA) and IAS-USA guidelines explicitly state available data does not support ART switch [3, 10]. Providers were increasingly comfortable using bictegravir/TAF/emtricitabine in pregnancy (15.1%, *n* = 18), although it is an alternative regimen in DHHS guidelines and IAS-USA guidelines [2, 3]. Finally, many providers (43.3%; *n* = 58) use lamivudine or emtricitabine in FDC despite renal dysfunction with a CrCl of < 30 mL/minute while not on hemodialysis. To our knowledge, this is one of the largest surveys of US HIV provider practice patterns and helps to characterize approaches when comorbidities or pregnancy are encountered.

PLHIV are at increased risk of cardiovascular events, compared to the general population [11]. Certain ART regimens have a more favorable lipid profile than others (i.e. TDF is associated with lower lipid levels than TAF; INSTIs and NNRTIs have fewer lipid effects than PIs) and an increased risk of cardiovascular events with abacavir has been observed in some studies but not all [2, 12, 13]. Furthermore, TAF and INSTIs may contribute to weight gain, which in turn could worsen cardiometabolic syndrome [14]. In our survey, providers chose to continue bictegravir/TAF/emtricitabine most frequently, but some altered their ART strategy, selecting a regimen that included TDF (*n* = 20, 10.5%) or was INSTI-sparing (14.7%, *n* = 28). As we understand more about cardiovascular risk in PLHIV, further data is needed to provide guidance on ART management strategies.

A signal for increased weight gain in PLHIV while on either TAF or a combination of a second generation INSTI (dolutegravir or bictegravir) and TAF has emerged [15–17]. US Guidelines either do not currently recommend ART switch when facing weight gain [3, 10] or provide flexibility to assess risks and benefits of regimens when initiating or changing ART, especially in Black women [2]. Notably in our survey, in case O3 detailing a 30-pound weight gain on dolutegravir plus TAF/emtricitabine, only 1 provider maintained the regimen, whereas 37.9% (*n* = 25) selected an INSTI and TAF-sparing regimen. A patient expressing concern for weight gain while initiating ART (Case O1), lead to 41.8% (*n* = 33) of providers to prescribe an NNRTI-anchor regimen (i.e. doravirine or rilpivirine), instead of an INSTI. Ultimately, it remains unknown if ART switch following weight gain will lead to weight loss, although a trial is ongoing evaluating doravirine for obese persons on integrase inhibitors and TAF (ClinicalTrials.gov Identifier: NCT04636437). Reductions in weight have been seen in some populations who switch from a TAF to TDF-containing regimen [18, 19]. A switch from an INSTI-containing regimen to a protease inhibitor did not show a significant change in weight at 24 weeks, however a trend towards weight loss was potentially seen with longer follow up [20]. Regardless, our survey demonstrated providers are weighing the potential contribution of TAF and INSTIs to weight gain and are often selecting alternative regimens.

In pregnancy, guidelines recommend initiation of a dolutegravir-based regimen with either tenofovir (TDF or TAF) plus emtricitabine or lamivudine, or dolutegravir/abacavir/lamivudine in certain situations. New to DHHS and IAS-USA guidelines, a bictegravir-containing regimen is an alternative initiating regimen for those trying to conceive or pregnant in the 1st or 2nd trimester [2, 3]. Bictegravir/TAF/emtricitaine is now indicated for use in pregnancy in the US Food and Drug Administration approved package insert (April 2024). Our surveyed providers initiated dolutegravir-containing regimens, however, in both cases combined, 15.1% (*n* = 18) started bictegravir/TAF/emtricitabine, potentially reflecting increasing comfort and preference for use in pregnancy. There continues to be emerging evidence supporting the use of bictegravir in pregnancy [21], although more data is needed.

The primary area of ambiguity surrounding ART and renal disease involves dosing of lamivudine or emtricitabine when CrCl decreases to < 30 mL/minute, with the US Food and Drug Administration approved package inserts recommending dose reductions in renal insufficiency [22, 23]. DHHS guidelines note that insufficient evidence exists for use of full-dose, daily lamivudine or emtricitabine if CrCl is < 15-30mL/minute but that some individuals will use full-dose to accommodate use of FDC [2]. In our survey, in two cases (R2 and R3) of patients with declining renal function on bictegravir/TAF/emtricitabine, 43.3% (*n* = 58) selected a regimen that contained FDC lamivudine or emtricitabine, and 14.2% (*n* = 19) still continued TAF. This demonstrates some provider willingness to dose ART outside of package insert recommendations, likely based on practical experience showing a lack of toxicity developing over many years of clinical experience and a desire to preserve FDC regimens.

This study does have limitations. We used hypothetical case-vignettes which may introduce biases that deviate from real-world practice patterns. Pragmatic approaches may also include shared-decision making that could not be simulated during an electronic survey. Our participants were primarily infectious diseases providers in large academic centers whose responses may not be generalizable to other clinician groups. Since the survey, the HIVMA has released new primary care guidance, and subsequent updates to DHHS guidelines have been made that may not be captured in this survey. For example, bictegravir/TAF/emtricitabine was recommended as an alternative regimen for pregnancy, which could lead to different survey responses if participants were queried today (i.e. an even higher proportion may favor its use). Furthermore, these responses may not be generalizable to all practicing HIV medicine, as primarily Infectious Diseases Specialists from academic institutions responded to our survey.

In conclusion, we sought to elucidate approaches to common comorbidities and co-occurring conditions seen in practice where ART management may not fit with the standard recommended initiating or treatment simplification regimens. Our responses provide insight into provider practice patterns and the survey responses could be used an educational tool for ART decision making tool for HIV providers when faced with co-occurring conditions in real life practice.

## Electronic supplementary material

Below is the link to the electronic supplementary material.

Supplementary Material 1

## Data Availability

The datasets used and/or analyzed during the current study are available from the corresponding author on reasonable request. Full responses to each case are available in the Supplementary Materials.
