# Supplementary material for: A United States HIV provider survey of antiretroviral therapy management in people living with HIV with co-occurring conditions
Source: AIDS Res Ther. 2025 Mar 1;22:27. doi: 10.1186/s12981-025-00724-w (PMC11871835; doi:10.1186/s12981-025-00724-w)
Supplement: Supplementary file 1 — Supplementary Material 1 [file 12981_2025_724_MOESM1_ESM.docx]

Supplementary Methods

Supplementary Table 1. Full list of antiretroviral abbreviations.

| Name | Abbreviation | Name | Abbreviation |
| --- | --- | --- | --- |
| Abacavir | ABC | Efavirenz | EFV |
| Bictegravir | BIC | Elvitegravir | EVG |
| Cabotegravir | CAB | Elvitegravir/cobicistat | EVG/c |
| Darunavir | DRV | Emtricitabine | FTC |
| Darunavir/cobicistat | DRV/c | Lamivudine | 3TC |
| Darunavir/ritonavir | DRV/r | Raltegravir | RAL |
| Darunavir/ritonavir twice daily | DRV/r BID | Rilpivirine | RPV |
| Dolutegravir | DTG | Tenofovir alafenamide | TAF |
| Dolutegravir twice daily | DTG BID | Tenofovir disoproxil fumarate | TDF |

Supplementary Table 2. Full clinical vignette by case.

| Case | Vignette |
| --- | --- |
| C1 | A 72-year-old male presents with a new diagnosis of HIV. He has a past medical history of coronary artery disease (CAD), diabetes mellitus (DM), hyperlipidemia, and obesity (BMI 31.1). He expresses concern about pill burden, possible side effects, and potential drug interactions.  HIV viral load: 55,000 copies/mL  CD4 count: 450 cells/mm3  Creatinine clearance (CrCl): >60 mL/min  Co-morbidities: CAD, DM, hyperlipidemia, Obesity  Co-medications: Aspirin, Lisinopril, Metoprolol, Metformin, Rosuvastatin  HLA-B5701: negative  Prior genotype: none  Current genotype: no mutations  Treatment history: none  Current Regimen: none  Tropism: unknown |
| C2 | A 58-year-old male is newly diagnosed with HIV. He has glucose intolerance (most recent HgA1c 6.2) and hypertriglyceridemia, being managed with diet. His most recent BMI was 31. He has a history of elevated transaminases and an ultrasound showing non-alcoholic steatohepatitis (NASH). He is negative for hepatitis B (HBV) and hepatitis C (HCV). His CD4 count is 174 and his viral load is 81,000.  HIV viral load: 81,000 copies/mL  CD4 count: 174 cells/mm3  Creatinine clearance (CrCl): >60 mL/min  Co-morbidities: NASH, transaminitis (AST 54, ALT 62, T bil 0.8, Alk Phos 150), hypertriglyceridemia, obesity  Co-medications: none  HLA-B5701: negative  Prior genotype: none  Current genotype: no mutations  Treatment history: none  Current Regimen: none  Tropism: unknown |
| C3 | A 70-year-old man who has sex with men (MSM) presents to your clinic after a recent diagnosis of HIV. He has a past medical history of hypertension (HTN), and hyperlipidemia (HLD). At the time of his visit, his CD4 count is 189 and his viral load is 79,210. He expresses concern about pill burden, possible side effects, and potential drug interactions.  HIV viral load: 79,210 copies/mL  CD4 count: 189 cells/mm3  Creatinine clearance (CrCl): >60 mL/min  Co-morbidities: HTN, HLD  Co-medications: Losartan, Hydrochlorothiazide, Amlodipine, Simvastatin  HLA-B5701: negative  Prior genotype: none  Current genotype: no resistance mutations  Treatment history: none  Current Regimen: none  Tropism: unknown |
| O1 | A 39-year-old female is newly diagnosed with HIV, following a a recent diagnosis of chlamydia cervicitis. She has a body mass index (BMI) of 29, has no other medical comorbidities, and does not have any plans to become pregnant (and is on oral combined hormonal contraceptives). The patient expresses concerns about any additional weight gain. Which regimen would you prescribe?  HIV viral load: 34,000 copies/mL  CD4 count: 450 cells/mm3  Creatinine clearance (CrCl): >60 mL/min  Co-morbidities: obesity  Co-medications: oral combined hormonal contraceptives  HLA-B5701: negative  Prior genotype: none  Current genotype: unknown  Treatment history: none  Current Regimen: none  Tropism: unknown |
| O2 | A 58-year-old female with a 15 year history of HIV and no known history of treatment failure presents as a new patient. Prior regimens have included Atripla (EFV/TDF/FTC) and DRV/r+TDF/FTC and she was suppressed on both. She has a 10 year history of type 2 diabetes mellitus (DM2), hypertension (HTN), hyperlipidemia (HLD), tobacco use (30 pack years), and a body mass index (BMI) of 30, having gained ~20lbs over the past 2 years. She is virally suppressed on her current regimen of Symtuza (DRV/c/TAF/FTC), which she has taken for three years, and has a CD4 count of 650. The patient is open to continuing the current regimen or changing the regimen based on your recommendation, and is open to injectable medications.  HIV viral load: <20 copies/mL  CD4 count: 650 cells/mm3  Creatinine clearance (CrCl): >60 mL/min  Co-morbidities: DM2, HTN, HLD, obesity, tobacco use  Co-medications: Metformin, Amlodipine, Losartan, Rosuvastatin  HLA-B5701: negative  Prior genotype: none  Current genotype: unknown  Treatment history: Atripla (EFV/TDF/FTC), DRV/r+TDF/FTC (suppressed on all prior regimens)  Current Regimen: Symtuza (DRV/c/TAF/FTC)  Tropism: unknown |
| O3 | A 52-year-old woman with a history of well-controlled HIV on Dolutegravir (DTG) + Descovy (TAF/FTC) presents for a follow up with a chief concern of a 30 pound weight gain over the course of 3 years while taking this regimen. Her baseline weight prior to treatment initiation was 160lbs (BMI 26.6) and her current weight is 190lbs (BMI 31.6). The patient is willing to change or continue the regimen based on your recommendation.  HIV viral load: <20 copies/mL  CD4 count: >200 cells/mm3  Creatinine clearance (CrCl): >60 mL/min  Co-morbidities: obesity  Co-medications: none  HLA-B5701: negative  Prior genotype: unknown  Current genotype:No resistance documented  Treatment history: Dolutegravir (DTG) + Descovy (TAF/FTC)  Current Regimen: Dolutegravir (DTG) + Descovy (TAF/FTC)  Tropism: unknown |
| P1 | A 31-year-old female presents with newly diagnosed HIV during an antenatal care visit. She is currently pregnant in her first trimester. Her CD4 is 195 and her viral load is 250,000.  HIV viral load: 250,000 copies/mL  CD4 count: 195 cells/mm3  Creatinine clearance (CrCl): >60 mL/min  Co-morbidities: none  Co-medications: none  HLA-B5701: negative  Prior genotype: none  Current genotype: no mutations  Treatment history: none  Current Regimen: none  Tropism: unknown |
| P2 | A 31-year-old female was diagnosed with HIV two years ago presents to establish care. She did not link to care after her initial diagnosis, but is sexually active with a new partner (HIV-uninfected) and she expresses interest in ART initiation now. She indicates she is interested in getting pregnant in the near future. Her CD4 is 195 and her viral load is 250,000.  HIV viral load: 250,000 copies/mL  CD4 count: 195 cells/mm3  Creatinine clearance (CrCl): >60 mL/min  Co-morbidities: preconception  Co-medications: none  HLA-B5701: negative  Prior genotype: none  Current genotype: No mutations  Treatment history: PrEP (TDF/FTC)  Current Regimen: none  Tropism: unknown |
| R1 | A 46-year-old man who has sex with men (MSM) with newly diagnosed HIV presents with a VL of 100,000 and CD4 >200. The patient has chronic kidney disease secondary to FSGS and is on hemodialysis.  HIV viral load: 100,000 copies/mL  CD4 count: >200 cells/mm3  Creatinine clearance (CrCl): <15mL/min, on hemodialysis  Co-morbidities: CKD stage 5, Hypertension  Co-medications: erythropoetin, vitamin d, sevelamer, iron, atenolol, amlodipine  HLA-B5701: negative  Prior genotype: none  Current genotype: no mutations  Treatment history: none  Current Regimen: none  Tropism: unknown |
| R2 | A 58-year-old male has a history of HIV, hypertension (HTN), poorly controlled type 2 diabetes mellitus (DM2), stage 2 chronic kidney disease (CrCl 80) and major depressive disorder (MDD). He has been virologically suppressed for greater than 12 months on his current regimen (BIC/TAF/FTC) and has a CD4 >200. On his current visit, he is noted to have worsening in his chronic kidney disease over the last year, with decline in >25% decline in eGFR and 1+ proteinuria on urinalysis. Serum and urine phosphate levels are normal. The patient is open to continuing the current regimen or changing the regimen based on your recommendation, including usage of injectable and long-acting medications.    HIV viral load: <20 copies/mL  CD4 count: >200 cells/mm3  Creatinine clearance (CrCl): 30-59 mL/min  Co-morbidities: CKD, HTN, DM2, MDD  Co-medications: Sertraline, Lisinopril, Metformin  HLA-B5701: negative  Prior genotype: no mutations  Current genotype: none  Treatment history: Biktarvy (BIC/TAF/FTC)  Current Regimen: Biktarvy (BIC/TAF/FTC)  Tropism: unknown |
| R3 | A 54-year-old female presents with a long history of HIV and a past medical history of chronic kidney disease (CKD). She has been suppressed for greater than 6 months on her current regimen of Biktarvy (BIC/TAF/FTC), but now presents with worsening renal function, which declined from CrCl 45 ml/min to <30 mL/min. The patient is open to continuing the current regimen or changing the regimen based on your recommendation, and is open to injectable medications.  HIV viral load: <20 copies/mL  CD4 count: >200 cells/mm3  Creatinine clearance (CrCl): 15-30 mL/min  Co-morbidities: CKD stage 4  Co-medications: none  HLA-B5701: negative  Prior genotype: no mutations  Current genotype: no mutations  Treatment history: Biktarvy (BIC/TAF/FTC)  Current Regimen: Biktarvy (BIC/TAF/FTC)  Tropism: unknown |

Supplementary Table 3. Full responses to case-vignettes with resistance.

| Case | Summary of Response by Regimen | n (%) |
| --- | --- | --- |
| C1 | BIC/TAF/FTC  DTG/3TC  DOR/TDF/3TC  RPV/TAF/FTC  DTG,TAF/FTC  DTG,TDF/FTC  DOR, TAF/FTC  DTG/RPV  FTC,3TC,DTG  TOTAL | 50 (62.50%) 13 (16.25%) 7 (8.75%) 3 (3.75%) 2 (2.50%) 2 (2.50%)  1 (1.25%)  1 (1.25%) 1 (1.25%)  80 |
| C2 | BIC/TAF/FTC  DTG/3TC  DOR/TDF/3TC  DRV/c/TAF/FTC  CAB/RPV  DOR, TAF/FTC  DTG, TAF/FTC  DTG/3TC,BIC/TAF/FTC  DTG/RPV  RPV/TAF/FTC  TOTAL | 22 (40.7%)  12 (22.2%)  10 (18.5%)  4 (7.4%)  1 (1.9%)  1 (1.9%)  1 (1.9%)  1 (1.9%)  1 (1.9%)  1 (1.9%)  54 |
| C3 | BIC/TAF/FTC  DTG/3TC  DOR/TDF/3TC  DTG/ABC/3TC  TOTAL | 47 (82.5%)  8 (24.0%)  1 (1.8%)  1 (1.8%)  57 |
| O1 | DOR/TDF/3TC  RPV/TAF/FTC  BIC/TAF/FTC  DRV/c/TAF/FTC  DTG/3TC  DTG/ABC/3TC  DOR, TAF/FTC  DTG, TDF/FTC  RAL,TAF/FTC  RPV/TDF/FTC  DTG,TAF/FTC  DTG/RPV, DTG/ABC/3TC, EVG/c/TAF/FTC  EVG/c/TAF/FTC  RAL,TDF/FTC  TDF/FTC, DRV/c TOTAL | 14 (17.7%)  13 (16.5%)  11 (13.9%)  9 (11.4%)  9 (11.4%)  6 (7.6%)  4 (5.1%)  4 (5.1%)  2 (2.5%)  2 (2.5%)  1 (1.3%)  1 (1.3%)  1 (1.3%)  1 (1.3%)  1 (1.3%)  79 |
| O2 | DRV/c/TAF/FTC  DOR/TDF/3TC  CAB/RPV  DTG/3TC  BIC/TAF/FTC  DTG/RPV  RPV/TAF/FTC  DTG,TDF/FTC  TOTAL | 13 (22.8%)  12 (21.1%)  11 (19.3%)  8 (14.0%)  5 (8.8%)  3 (5.3%)  3 (5.3%) 2 (3.5%)  57 |
| O3 | DOR/TDF/3TC  DRV/c/TAF/FTC  DTG/3TC  RPV/TAF/FTC  RPV/TDF/FTC  DTG/RPV  BIC/TAF/FTC  CAB/RPV  DOR,TAF/FTC  DTG,TDF/FTC  DTG/ABC/3TC  DOR, DRV/c  DOR, DTG  DOR,TDF/FTC  DRV/r,TDF/FTC  DTG,TAF/FTC  UNABLE TO ANSWER  OTHER  TDF/FTC,DRV/c  TOTAL | 17 (25.8%)  11 (16.7%)  7 (10.6%)  6 (9.1%)  4 (6.1%)  3 (4.6%)  2 (3.0%)  2 (3.0%)  2 (3.0%)  2 (3.0%)  2 (3.0%)  1 (1.5%) 1 (1.5%)  1 (1.5%) 1 (1.5%) 1 (1.5%) 1 (1.5%) 1 (1.5%)  1 (1.5%)  66 |
| P1 | DTG,TAF/FTC  DTG/ABC/3TC  DTG,TDF/FTC  BIC/TAF/FTC  RAL/TDF/FTC  RPV/TAF/FTC  DRV/r BID, TDF/FTC  DRV/r,TDF/FTC  DTG,DRV/r,TDF/FTC  FTC,TAF,DTG  TAF,DTG  TOTAL | 22 (33.3%)  14 (21.2%)  12 (18.2%)  9 (13.6%)  2 (3.0%)  2 (3.0%)  1 (1.5%)  1 (1.5%)  1 (1.5%)  1 (1.5%)  1 (1.5%)  66 |
| P2 | DTG,TAF/FTC  DTG/ABC/3TC  BIC/TAF/FTC  DTG, TDF/FTC  DTG BID,TAF/FTC  DTG/3TC  RAL,TDF/FTC  DRV/c/TAF/FTC  DRV/r,TDF/FTC  FTC,TAF,DTG  RPV/TAF/FTC  TAF,DTG  TOTAL | 15 (28.3%)  12 (22.6%)  9 (17.0%)  6 (11.3%)  2 (3.8%)  2 (3.8%)  2 (3.8%)  1 (1.9%)  1 (1.9%)  1 (1.9%)  1 (1.9%)  1 (1.9%)  53 |
| R1 | BIC/TAF/FTC  DTG/3TC  DTG/ABC/3TC  EVG/c/TAF/FTC  3TC,TDF,DTG  DOR,DTG  ABC,3TC,DTG  TDF,DTG/3TC  3TC,DOR,DTG  3TC, DTG  3TC,DTG,DRV/r  ABC,FTC,DTG  DRV/c/TAF/FTC  DTG,DRV/c  DTG,TAF/FTC  DTG,RPV  DTG/RPV,DRV/c  FTC,TDF,DTG  TOTAL | 21 (38.9%)  5 (9.3%)  4 (7.4%)  4, (7.4%)  3, (5.6%)  3, (5.6%)  2, (3.7%)  2 (3.7%)  1 (1.9%)  1 (1.9%) 1 (1.9%) 1 (1.9%) 1 (1.9%) 1 (1.9%)  1 (1.9%)  1 (1.9%)  1 (1.9%)  1 (1.9%)  54 |
| R2 | DTG/3TC  CAB/RPV  DTG/RPV  BIC/TAF/FTC  3TC,DTG  DTG/ABC/3TC  TOTAL | 18 (31.6%)  15 (26.3%)  12 (21.1%)  10 (17.5%)  1 (1.8%)  1 (1.8%)  57 |
| R3 | DTG/RPV  CAB/RPV  DTG/3TC  DTG/ABC/3TC  BIC/TAF/FTC  ABV,3TC,DTG  3TC,DTG  3TC, DTG BID  DOR,DTG  DTG,DRV/c  EVG/c/TAF/FTC  TOTAL | 24 (31.2%) 12 (15.6%)  11 (14.3%)  9 (11.7%)  8 (10.4%)  6 (7.8%)  3 (3.9%)  1 (1.3%)  1 (1.3%)  1 (1.3%)  1 (1.3%)  77 |
